# A Rapid, Scalable Method for the Isolation, Functional Study, and Analysis of Cell-derived Extracellular Matrix

**DOI:** 10.3791/55051

**Published:** 2017-01-04

**Authors:** Andrew L. Hellewell, Silvia Rosini, Josephine C. Adams

**Affiliations:** ^1^School of Biochemistry, University of Bristol

**Keywords:** Cellular Biology, Issue 119, extracellular matrix, isolation, ammonium hydroxide, live-cell imaging, proteomics, fluorescent protein tag

## Abstract

The extracellular matrix (ECM) is recognized as a diverse, dynamic, and complex environment that is involved in multiple cell-physiological and pathological processes. However, the isolation of ECM, from tissues or cell culture, is complicated by the insoluble and cross-linked nature of the assembled ECM and by the potential contamination of ECM extracts with cell surface and intracellular proteins. Here, we describe a method for use with cultured cells that is rapid and reliably removes cells to isolate a cell-derived ECM for downstream experimentation.

Through use of this method, the isolated ECM and its components can be visualized by *in situ* immunofluorescence microscopy. The dynamics of specific ECM proteins can be tracked by tracing the deposition of a tagged protein using fluorescence microscopy, both before and after the removal of cells. Alternatively, the isolated ECM can be extracted for biochemical analysis, such as sodium dodecyl sulphate-polyacrylamide gel electrophoresis (SDS-PAGE) and immunoblotting. At larger scales, a full proteomics analysis of the isolated ECM by mass spectrometry can be conducted. By conducting ECM isolation under sterile conditions, sterile ECM layers can be obtained for functional or phenotypic studies with any cell of interest. The method can be applied to any adherent cell type, is relatively easy to perform, and can be linked to a wide repertoire of experimental designs.

**Figure:**
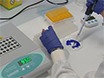


## Introduction

Over the last few decades, the extracellular matrix (ECM) has become recognized as a diverse, dynamic, and complex environment that is involved in multiple cell-physiological and pathological processes. At the tissue level, the ECM influences cell signaling, motility, differentiation, angiogenesis, stem cell biology, tumorigenesis, fibrosis, *etc.*^1,2^. The study of ECM organization and ECM-dependent processes thus has wide implications for cell biology and tissue physiology. To reach a mechanistic understanding of ECM composition, organization, and functional properties, methods for the accurate isolation of ECM are required. Whereas the earliest identification of ECM proteins relied upon isolation from tissues^3^, the preparation of ECM from cultured cells has now become more prevalent.

The isolation and analysis of cell-derived ECM is complicated for two main reasons. Firstly, the presence of cells and their abundant intracellular proteins can make it difficult to isolate the ECM as a discrete extracellular structure. Indeed, some ECM proteins have roles inside the cell as well as in the ECM^4^; therefore, the efficient removal of intracellular proteins from cell-derived ECM is vital if the study of proteins within the ECM is not to be confused with their roles inside the cell. Secondly, cell-derived ECM is composed of many large, oligomeric proteins, which are often covalently cross-linked upon ECM assembly and are therefore insoluble in standard detergents. These properties can complicate extraction and the further analysis of ECM. To address these issues, a method is required that enables the efficient separation of ECM proteins from cellular components.

Several methods have been described in the literature for the isolation of ECM from either cell culture or tissue extracts. Many of these methods are aimed at the extraction of the abundant ECM protein, collagen, from tissues and include the use of neutral salts^3^, acidic conditions^5,6^, or pepsin^7^. Isolation of total ECM from tissue extracts often involves decellularization of the tissue prior to the isolation of the ECM. For example, a sequential extraction method has been described that isolates ECM from human cardiac tissue^8^. First, loosely-bound ECM proteins were extracted with 0.5 M NaCl before sodium dodecyl sulphate (SDS) was used to remove the cells. Finally, the remaining ECM proteins were extracted using 4 M guanidine^8^. ECM can also be solubilized from decellularized samples using 8 M urea^9^. Other techniques use detergents, such as deoxycholic acid^10^, to extract both cells and ECM before separating insoluble ECM proteins from the soluble cell lysate by centrifugation.

The method for isolation and analysis of cell-derived ECM described in this report provides a reproducible method for removing cell material, leaving cell-derived ECM that can be analyzed by* in situ* immunofluorescence or extracted for further biochemical analysis. This method can be adapted for any adherent cell type and can be scaled up for downstream procedures, such as immunoblotting or mass spectrometry, or for utilization of the isolated ECM in functional studies. The method can also be used in conjunction with confocal microscopy of live cells to track ECM deposition of a tagged protein of interest in real time. This is achieved through the use of a gridded, glass-bottomed dish. Overall, the approach provides an accurate isolation of cell-derived ECM and also the scope to identify and monitor the deposition and dynamics of individual ECM proteins.

## Protocol

### 1. Removal of Cells with Ammonium Hydroxide Solution

1. Prepare Adherent Cells by Plating at the Appropriate Density. NOTE: The cells can be any adherent cell type that produces sufficient ECM for analysis. Here, we describe the use of COS-7 cells, an African green monkey kidney fibroblast-like cell line that contains SV-40 viral DNA sequences; RCS, a rat chondrosarcoma cell line; or normal human dermal fibroblast (HDF) strains from juvenile foreskin. HDF are used from passage 1 to passage 8 only.
  1. Plate the cells on coverslips for the imaging of live cells and ECM, for fluorescence microscopy studies of fixed ECM, or for preparation of cell-derived ECM for small-scale functional assays. Plate the cells on cell culture dishes for SDS-PAGE analysis, immunoblot, or proteomics studies. NOTE: The cell culture conditions (number of cells to plate and culture medium) will depend on the cell type. The cell number to plate will need to be established empirically for the particular cell line or cell strain.
  2. Allow a suitable time for the cells to deposit ECM, typically >16 h. NOTE: The time period will depend on the cell type and will need to be determined empirically. If conducting ectopic expression, allow appropriate time for the expression of the transfected protein of interest and for the deposition of ECM.
2. In an Extractor Hood, Prepare 20 mM Ammonium Hydroxide in a Suitable Vessel by Diluting the Stock Solution 1/14 with De-ionized H_2_O.
  1. Remove the cells from the incubator and gently remove culture medium. Add phosphate-buffered saline (PBS) without Ca^2+^/Mg^2+ ^by gently pouring against the walls of the dish. Rock the dish twice and remove the liquid with a plastic transfer pipette. Repeat twice more.
3. In an extractor hood, tilt each dish and remove the PBS from step 1.2 with a plastic transfer pipette. Add 3 mL of ammonium hydroxide per 100-mm dish and incubate them at room temperature for 5 min. During the 5-min incubation period, gently agitate the dish every min to ensure the lysis of all the cells. Steps 1.4-1.7 will also be carried out in the extractor hood. NOTE: Alternative cell removal reagents include 2 M or 8 M urea, which are incubated with the cells for 10 min.
4. Add copious amounts of de-ionized H_2_O to each dish, at least 20 mL per 100 mm dish, with rocking. Dispose of the ammonium hydroxide-solubilized material-which is composed of ammonium hydroxide, lysed cells, and de-ionized H_2_O-by aspiration with a transfer pipette. Transfer this waste solution into a container for liquid waste.
5. Wash the insoluble ECM layer in copious de-ionized H_2_O four more times to ensure the complete removal of all the ammonium hydroxide-solubilized material.
6. For examination of the ECM by immunofluorescence, fix the ECM by adding 2% paraformaldehyde (PFA; 3 mL per 100 mm dish) at room temperature for 10 min. Caution: PFA is very hazardous in case of skin or eye contact and severe overexposure can produce lung damage, choking, unconsciousness, or death. It should only be handled in an extractor hood, and personal protective equipment should be worn.
7. Wash each fixed sample twice with PBS, as in step 1.2, and dispose of the PFA solution into a suitable liquid waste container. If necessary, store the ECM samples in PBS at 4 °C before preparing them for fluorescence microscopy.

### 2. Tracking Deposition of an ECM Protein by Confocal Microscopy Imaging of Live Cells

1. Count the cells under a hemocytometer. For COS-7 cells, plate 250,000 cells onto a 60 mm cell culture dish for transfection. NOTE: For live cell imaging, the cells must be transfected with a vector encoding the ECM protein of interest, fused in frame with a genetically-encoded fluorescent tag. This is to enable the identification of the ECM protein of interest during live-cell imaging.
2. After a suitable time for protein expression (*e.g.,* 16 h) remove the cells from the dish with trypsin-EDTA solution, count them in a hemocytometer, and plate ~150,000 cells onto a 35-mm gridded, glass-bottomed dish for imaging. Allow 2 h for the cells to reattach and to begin ECM deposition (the time period will depend on the cell type and must be established empirically). NOTE: Use a cell medium that does not contain phenol red, as this can give a red tinge that affects image quality.
3. During the above 2 h period, set up a confocal microscope with a 37 °C incubator chamber and a CO_2_ supply. Allow the temperature in the chamber and in the dish to stabilize to 37 °C before imaging; this can take up to 1 h.
4. Incubate the cells plated onto the 35 mm gridded dish with a fluorescent cell marker for 30 min. Then, wash the cells twice in phenol red-free culture medium prior to imaging. NOTE: A cytoplasmic fluorescent marker can be used to visualize cell volumes, or a membrane-incorporating marker can be used to visualize cell edges.
5. Using the 20X objective and phase contrast on a confocal microscope, identify an appropriate grid square that contains a number (>3) of adherent, healthy cells. Confirm the expression of the target protein of choice in the cells within the selected grid square using the 63X or 100X objectives and the appropriate wavelengths under fluorescence microscopy.
6. Set up fluorescence and phase-contrast time-lapse imaging using a z-stack software. Set the "Begin" stack to be just below the ECM layer and the "End" stack to be just above the cell body. Set the z-step size to 0.3 µm and the z-volume to between a 5 and 10 µm depth in total, depending on the cell type. This will give between 15-30 z-sections per time point.
7. Capture fluorescence (for red fluorescent protein (RFP), excitation = 555 nm and emission = 584 nm) and phase-contrast images periodically over a set time period. For example, use the time-lapse software to set the time interval at 2 min and the duration to 2 h. Select the "start" button to begin capture of z-section images over the defined time period.
8. At the end of the time period, remove the cells from dish, as described in steps 1.1-1.5, to confirm the localization of the fluorescently-tagged protein of interest in the ECM.
9. Use phase-contrast imaging and the 20X objective to relocate the relevant grid square.
10. Switch to the 63X objective and identify the ECM layer under fluorescence microscopy. Compare this image to the pre-extraction image

### 3. Sterile Preparation of Cell-derived ECM for Cell Functional Assays

1. Grow one population of cells (the "matrix producer" cells) on coverslips and another population (the "test" cells) in standard culture in a 100 mm cell culture dish for at least 24 h. NOTE: These can be any two different adherent cell types, and the experimental design can include transfection of one or both cell populations to express a fluorescently-tagged ECM protein.
2. In a laminar flow hood, as used for cell culture, prepare sterile solutions of PBS without Ca^2+^/Mg^2+^, 20 mM ammonium hydroxide (see step 1.3), and de-ionized H_2_O by passing each through a sterile 0.2 µm filter into an appropriate sterile container.
3. Maintaining sterile technique, gently wash the "matrix producer" cells on coverslips three times with sterile PBS (see step 1.2).
4. Remove the PBS by aspiration with a sterile pipette and dispose it in a suitable liquid waste container. Add 20 mM sterile ammonium hydroxide (3 mL/100 mm dish) and incubate them for 5 min with rocking at intervals.
5. Add copious amounts of sterile de-ionized H_2_O to the "matrix producer" dish, at least 20 mL per 100 mm dish, with rocking, and pour away the cell lysate into a suitable waste container.
6. Repeat step 3.5 four more times to ensure the complete removal of the ammonium hydroxide-solubilized material. NOTE: The ECM deposited by the "matrix producer" cells on the coverslips then provides a sterile ECM for functional assays with any test cells of interest.
7. Incubate the test cells from the cell stock dish with 1.5 mL of trypsin-EDTA solution per 100 mm dish (0.05% w/v trypsin and 0.02% w/v EDTA in Hank's balanced salt solution) until the test cells are detached from the dish. Add cell medium, centrifuge the test cells at 400 x g for 5 min, and remove the supernatant liquid.
8. Resuspend the test cells in fresh, sterile medium and count them in a hemocytometer. Plate an appropriate number of these test cells onto the sterile, isolated ECM on coverslips. Culture them in the appropriate cell medium for 2-3 h, or for the time period required for the experimental design.
9. To examine the organization of the actin cytoskeleton, fix the test cells in 2% PFA and stain them with fluorescein isothiocyanate (FITC)-phalloidin (excitation = 490 nm and emission = 525 nm) to visualize F-actin and with 4',6-diamidino-2-phenylindole (DAPI; excitation = 358 nm and emission = 461 nm) to visualize the nuclei^11^. NOTE: Compare the morphology and actin organization in the test cells plated on heterologous cell-derived ECM with test cells plated on their own ECM.

### 4. Isolation of ECM for SDS-PAGE, Immunoblot Analysis, or Proteomics

1. Grow adherent cells of interest on 100 mm cell culture dishes (5 to 10 dishes for proteomics or 1-2 dishes for immunoblotting) for 24-96 h, as appropriate for the cell type, to allow secretion and deposition of ECM.
2. Remove the cells as described in steps 1.1-1.5.
3. Tilt each plate at an angle to drain residual H_2_O to the bottom of the dish, and carefully remove any remaining H_2_O with a p200 pipet until the plate is completely dry.
4. In parallel, heat SDS-PAGE sample buffer containing 100 mM dithiothreitol (DTT) to 95 °C for 2 min using a heat block, and then add it to the plate. 200 µL of sample buffer per 100 mm plate is appropriate for samples to be examined by immunoblot.
  1. To analyze ECM by mass spectrometry, achieve a more concentrated sample by collecting the SDS-PAGE sample buffer from the dish (as described in steps 4.5 and 4.6, below), re-heating it to 95 °C, and using the same aliquot across 2-3 additional plates.
5. Use a cell scraper to thoroughly scrape the ECM off the dish, ensuring that all areas of the dish have been scraped.
6. With the cell scraper, gather the sample to one side of the dish and remove it with a 200 µL pipette tip into a tube.
7. Transfer any residual SDS-PAGE sample buffer extract from the cell scraper tip into the tube to minimize the loss of ECM material. For a concentrated sample, re-heat the same SDS-PAGE sample buffer aliquot to 95 °C and re-use it across 2-3 additional plates.
8. Resolve the ECM proteins by SDS-PAGE^12^, using either 7% or 4-7% gradient polyacrylamide gels. NOTE: It is convenient to use pre-stained protein markers.
9. To increase the resolution of the high-molecular-weight ECM proteins, extend the gel running time such that the 37 kDa molecular weight marker runs close to the bottom of the gel and molecular weight markers <37 kDa have run off the gel.
10. For proteomic analysis of ECM proteins, stain the gel with a protein stain and capture an image. Cut bands of interest from the gel, digest them with trypsin, and analyze them by matrix-assisted laser desorption/ionization (MALDI)-mass spectrometry^13^.
11. Alternatively, for a more comprehensive analysis of the ECM extract and to analyze the entire sample, slice the gel lane into sections, digest them with trypsin, and perform liquid chromatography-mass spectrometry (LC-MS)^14^.
12. Alternatively, for immunoblotting^15^, transfer proteins from the SDS-PAGE gel onto polyvinylidene fluoride (PVDF) membrane at 15 V for at least 1 h 10 min (semi-dry method) to ensure the transfer of the high-molecular-weight ECM proteins. Visualize the total protein transferred to the membrane with a Ponceau S stain.
13. Following step 4.11, use antibodies to establish the presence and/or make a semi-quantitative analysis of individual ECM proteins^15^.
  1. Block the membrane with 2% (w/v) milk in TBS-Tween 20 (blocking buffer) overnight at 4 °C. Dilute specific antibodies to ECM proteins in blocking buffer and incubate with the membrane for 1.5 h at room temperature (antibody to fibronectin diluted 1/600 and antibody to thrombospondin-1 diluted 1/150; **Supplementary Table 1**).
  2. Test the quality of the ECM isolation by re-probing the same blot with antibodies to abundant intracellular proteins, such as actin or tubulin (anti-actin diluted 1/10,000 and anti-tubulin diluted 1/5,000).

## Representative Results

The protocol described in steps 1.1-1.5 acts to remove cells and leave behind the cell-derived ECM. The protocol was carried out on COS-7 cells grown for 96 h on glass coverslips, and the cell-derived ECM was analyzed by fluorescence microscopy. The ammonium hydroxide treatment removed the cells effectively, as established by the loss of the cell nuclei and actin cytoskeleton, according to DAPI and phalloidin staining, respectively (**Figure 1**). The extraction of cells with 2 M urea was not as effective as ammonium hydroxide; traces of DAPI and phalloidin staining were detected after treatment. 8 M urea had a similar effect to ammonium hydroxide (**Figure 1**). Next, cell-derived ECM was visualized before and after the ammonium hydroxide treatment (steps 2.1-2.11). COS-7 cells were transfected with a monomeric red fluorescent protein (mRFP)-tagged thrombospondin-1 C-terminal trimer, (mRFPovTSP1C)^11^ and grown on a 35 mm dish with a gridded glass base.

Two hours post-plating, a suitable grid square that contained multiple healthy cells expressing mRFPovTSP1C was visualized under a confocal microscope. A reference phase contrast image was taken of this area, and then fluorescence time-lapse imaging was carried out every 1 min for 2 h (**Figure 2A**). Cells were then removed using ammonium hydroxide, and the same grid square on the dish was relocated under phase contrast and then re-analyzed by fluorescence microscopy. The cells were no longer present in the grid square, but mRFPovTSP1C remained within the ECM, detected by fluorescence microscopy as characteristic puncta (**Figure 2A**). Any mRFPovTSP1C deposited in the ECM prior to cell removal was identified by comparing the fluorescence pattern pre- and post-removal of cells. The fluorescent puncta identified in both images (**Figure 2A**, example arrowed) are inferred to be associated with the ECM.

Ammonium hydroxide treatment was then used to isolate native ECM from cultured, adherent cells. Human dermal fibroblasts (HDF) were grown on glass coverslips for 96 h. Cells were removed using ammonium hydroxide, and the ECM was probed with an anti-collagen I antibody, which demonstrated a characteristic fibrillar and meshwork staining pattern^16^ (**Figure 2B**). Fibronectin patterning was examined around fixed, non-permeabilized rat chondrosarcoma cells (RCS) and within the ECM after the removal of the RCS cells (**Figure 2C**). The ammonium hydroxide isolation of ECM was also carried out under sterile conditions so that "test" cells could be re-plated onto the ECM for phenotypic or functional studies. For example, "test" COS-7 cells were plated for 2 h onto sterile ECM produced by RCS cells, and then they were fixed, permeabilized, and analyzed for F-actin organization by FITC-phalloidin staining, in comparison to COS-7 cells producing their own ECM (steps 3.1-3.9) (**Figure 3**).

At a larger scale, the method was used to isolate ECM for biochemical procedures. For example, RCS cells were grown on two 100 mm cell culture dishes for 7 days, and the procedures in steps 4.1-4.7 were followed. Proteins in the cell-derived ECM were collected by scraping them into hot SDS-PAGE sample buffer and were separated by SDS-PAGE on a 7% polyacrylamide gel under reducing conditions. The gel was stained for protein, and the four major bands were each isolated and analyzed by mass spectrometry (**Figure 4A**). A number of ECM proteins, including fibronectin, thrombospondin 1 (TSP1), thrombospondin 5/cartilage oligomeric matrix protein (TSP5 / COMP), and matrilin-1 were identified (**Figure 4B**).

Alternatively, smaller-scale preparations of ECM can be evaluated by immunoblots. For example, cell-derived ECM from COS-7 cells ectopically expressing mRFPovTSP1C was separated by SDS-PAGE, transferred to a PVDF membrane, and probed for RFP with an anti-RFP antibody (**Figure 4C**). This technique is sensitive enough to detect differences in deposition between a native trimer of TSP1 (mRFPovTSP1C) and an engineered pentamer of the TSP1 C-terminal region (mRFP-TSP-5-1C)^17^ (**Figure 4C**). To investigate the endogenous ECM of HDF, the presence of specific proteins in cell lysate or the isolated ECM was examined by immunoblotting. The characteristic ECM proteins fibronectin and thrombospondin-1 were detected in the ECM, whereas the abundant intracellular proteins α-tubulin and β-actin were absent from the isolated ECM (**Figure 4D**).


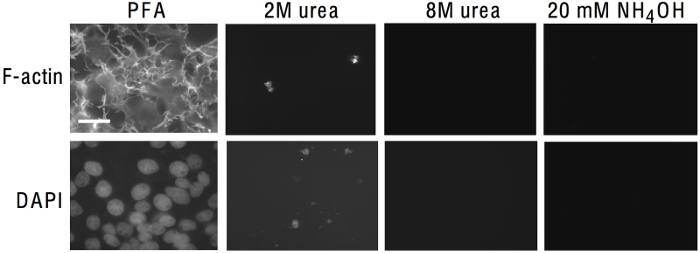
**Figure 1: Comparison of Methods of Cell Removal. **COS-7 cells were grown at high density for 96 h on coverslips, fixed in 2% PFA, and permeabilized in 0.5% Triton X100, or were treated as indicated for cell removal. Coverslips were then stained with FITC-phalloidin to visualize F-actin and DAPI to visualize nuclei. Scale bar = 25 µm. This figure is modified from an original publication in the Journal of Cell Science^11^. Please click here to view a larger version of this figure.


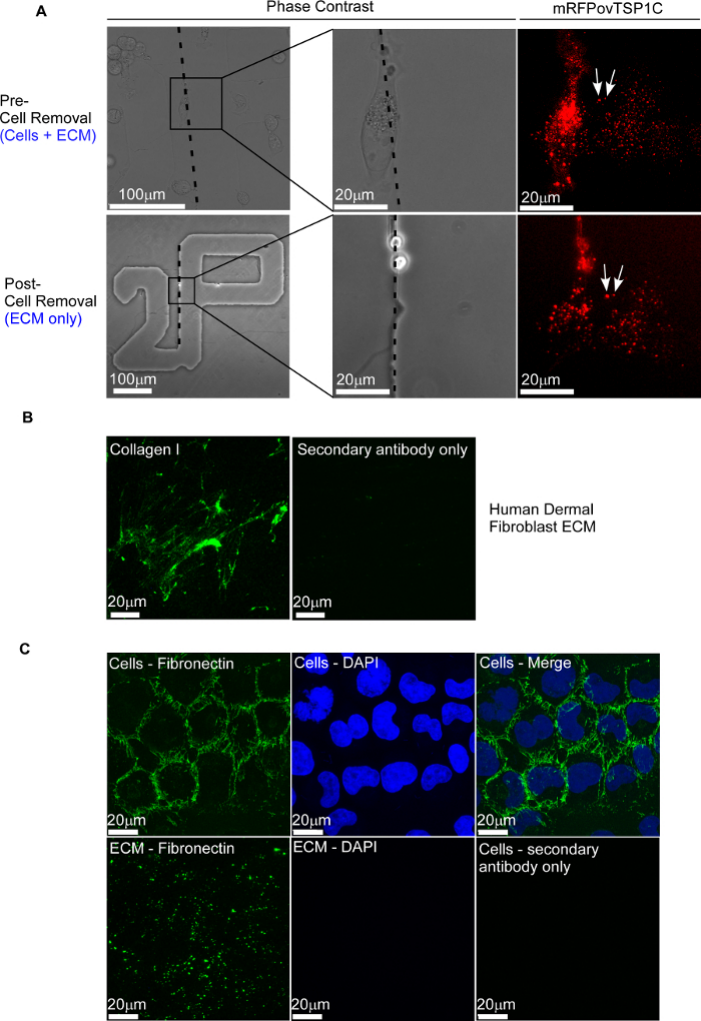
**Figure 2:Identification of ECM Proteins in Isolated ECM. **(**A**) Phase-contrast and fluorescence images of COS-7 cells expressing mRFPovTSP1C and grown on a 35 mm dish with a glass-bottomed, gridded base for 2 h. Images are shown before and after the removal of the cells by ammonium hydroxide. The edge of the grid letter is indicated in the phase-contrast images with a dotted line. White arrows indicate examples of fluorescent puncta that were present before and after cell removal. (**B**) Detection of collagen I in HDF ECM by indirect immunofluorescence. HDF were grown for 96 h and removed with ammonium hydroxide, and the ECM was stained for human fibrillar collagen I. The ECM was also stained with FITC-conjugated anti-rabbit secondary antibody alone. (**C**) Localization of fibronectin around RCS cells or within isolated RCS ECM, as detected by indirect immunofluorescence. RCS cells were grown for 48 h, fixed in 2% PFA, and stained for fibronectin and with DAPI. In parallel dishes, cells were removed with 20 mM ammonium hydroxide and the ECM was stained for fibronectin and with DAPI. Cells were also stained with FITC-conjugated anti-mouse IgG antibody alone. Please click here to view a larger version of this figure.


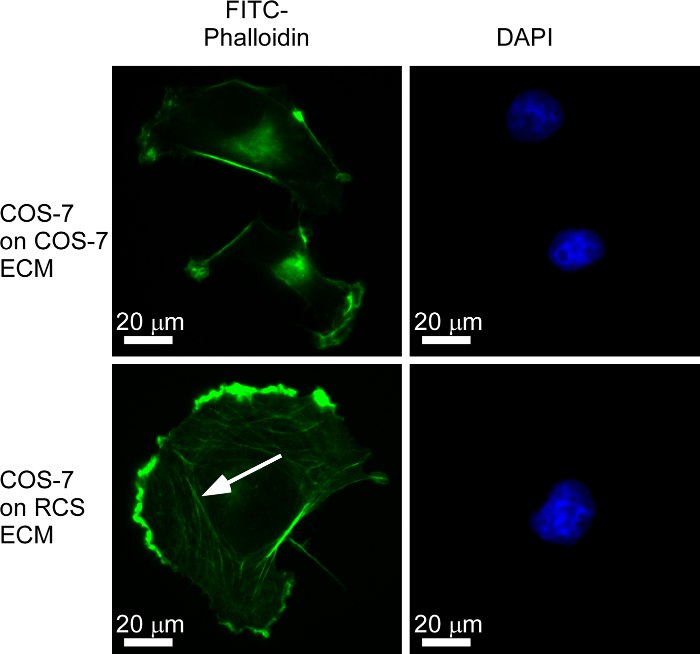
**Figure 3:Use of Sterile ECM for Functional Studies. **RCS "matrix producer" cells were grown for 48 h on glass coverslips and then treated with 20 mM ammonium hydroxide under sterile conditions to remove the cells. COS-7 "test" cells were plated on coverslips with or without isolated RCS ECM for 2 h, fixed in 2% PFA, permeabilized in 0.5% Triton X100, and stained with FITC-phalloidin to visualize F-actin and DAPI to visualize nuclei. COS-7 cells plated on RCS ECM spread more extensively and formed large microfilament bundles (example arrowed) and edge ruffles. Please click here to view a larger version of this figure.


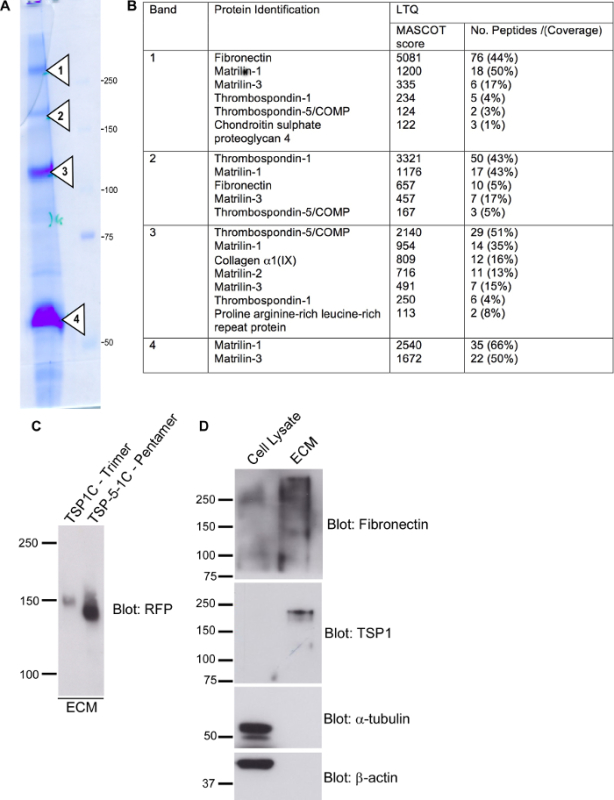
**Figure 4:Identification of Proteins from Isolated ECM by SDS-PAGE, Mass Spectrometry, or Immunoblotting. **(**A**) RCS cells were grown for 7 days and removed with 20 mM ammonium hydroxide; the ECM was scraped up into hot SDS-PAGE sample buffer containing 100 mM DTT. The ECM preparation was separated by SDS-PAGE on a 7% polyacrylamide gel under reducing conditions. Four significant bands (arrows 1-4) were identified by protein staining. (**B**) Proteins from RCS ECM bands 1-4 were identified by MALDI mass spectrometry. (**C**) COS-7 cells expressing either mRFPovTSP1C (trimer) or mRFP-TSP-5-1C (pentamer) were cultured for 48 h and removed with 20 mM ammonium hydroxide, and the was ECM isolated into hot SDS-PAGE sample buffer containing 100 mM DTT. The ECM preparation was separated by SDS-PAGE on a 7% polyacrylamide gel under reducing conditions, transferred to a PVDF membrane, and probed with an anti-RFP antibody. This panel is modified from an original publication in Bioscience Reports^17^. (**D**) HDF were grown for 96 h and then treated either with 2% deoxycholic acid in PBS (cell lysate) or with 20 mM ammonium hydroxide to remove cells. The ECM was scraped into hot SDS-PAGE sample buffer. The two fractions were separated by SDS-PAGE on a 10% polyacrylamide gel under reducing conditions, transferred to a PVDF membrane, and probed with antibodies, as indicated. In each panel, the positions of molecular weight markers are indicated in kDa. Please click here to view a larger version of this figure.

## Discussion

### Critical Steps within the Protocol

The method has some critical steps that must be followed to ensure the successful isolation and analysis of ECM. For example, ammonium hydroxide is used to remove the cells and to isolate ECM for analysis. Although ammonium hydroxide is stable in aqueous solutions in the dark and can be stored at room temperature, bottles must be tightly re-sealed between each use. Because the gas may evaporate from the solution, thus reducing the concentration, we strongly recommend starting a fresh bottle every 6-8 weeks. In addition, the working solution should be made freshly for each experiment. It is also essential that the cells are fully removed with copious washes in de-ionized water. If these steps are not performed adequately, cellular material may remain on the dish and contaminate downstream analyses. If cells have been grown for 10 days or longer, additional water washes may be needed. It is important to note that the isolated ECM layer is typically very thin in comparison to the cells^11^, so care is needed when identifying the ECM during imaging. For effective extraction of the isolated ECM into SDS-PAGE sample buffer, it is essential that the sample buffer contains a reducing agent. For the most effective removal of the ECM, boiling-hot sample buffer must be used. For experiments in which ECM samples will be resolved by SDS-PAGE electrophoresis for protein staining or proteomics, it is critical to achieve a concentrated sample by scraping several plates-worth of ECM into the same aliquot of sample buffer.

### Modifications and Troubleshooting

The production and secretion of ECM proteins can vary significantly between cell types, so time periods for secretion and deposition of ECM should be determined *de novo* for each cell type. It is also important to be aware that ECM composition will change dynamically in relation to cell density and time in culture^18^. This factor should be taken into account when designing experiments. Clearly, the selection of a cell type for this method is important, particularly when extracting ECM for analysis by mass spectrometry, which may require a substantial amount of total ECM protein.

### Limitations of the Technique

Cells that secrete very low levels of ECM proteins will be more challenging for use with this method. Non-adherent cells, 3D cell cultures, or cell invasion assays are unsuitable at present for ECM isolation by this method. The method is most suitable for use with standard "2-dimensional" cell cultures. Because the ammonium hydroxide extraction removes the cells completely, ECM associated with the upper sides of cells and secreted, but non-cross-linked, ECM proteins are also removed, leaving on the dish a thin layer of assembled ECM from below and around the bases of the cells^11,17^. It is complex to quantify how much ECM is released by the ammonium hydroxide extraction, because the released material also includes ECM proteins that are intracellular either prior to secretion or after uptake from the cell surface.

### Significance of the Technique with Respect to Existing/Alternative Methods

The ability to conduct a wide range of experiments with isolated ECM is important in the context of cell biology and tissue physiology, and it also has implications for the fields of tissue regeneration and tissue engineering. The method has advantages over gels formed from purified collagens or other purified ECM proteins in that the cells assemble complex ECM structures at their native size, with native cross-linking mechanisms and with individual ECM proteins present in ratios appropriate to the cell type of origin. For example, the proteomic study of RCS ECM demonstrated abundant matrilins and COMP/TSP5, as expected for a cartilaginous ECM^19,20^ (**Figure 4**). In general, the analysis of cell-derived ECM is hampered by its nature as an extensive, multi-protein network that results in covalent crosslinking and the insolubility of many ECM proteins, and also by the potential contamination of ECM extracts with intracellular proteins. A multi-step procedure designed to isolate the ECM fraction from tissues for proteomic analysis yielded 8% of ECM proteins in the total set of proteins identified^21^. However, peptides from ECM proteins were 73% of the total peptides identified. This method for isolation and analysis of cell-derived ECM rapidly and reliably removes cellular material whilst retaining ECM proteins. This method can be used for a range of cell types and downstream applications and facilitates the analysis of ECM biology and cell-ECM interactions in cell culture. Our protocols detail how the method can be applied at different scales to address a wide range of experimental questions with regard to ECM organization, dynamics, or composition, and to the functional effects of isolated ECM on cells.

### Future Applications or Directions after Mastering This Technique

Looking to the future, the method could be adapted for use with 3D cell cultures; this would expand its physiological relevance. Decellularized native tissue has great potential as a scaffold for the regeneration of lost or damaged tissue and as an alternative to transplantation, such as after heart failure^22^. It has the potential to overcome the problems of donor availability and immunorejection. Future applications of the method described in this report could investigate its use with 3D cell cultures or tissue decellularization, which would further increase the scope of this approach.

## Disclosures

The authors declare that they have no competing financial interests.
